# Peripapillary retinal artery in first diagnosed and untreated normal tension glaucoma

**DOI:** 10.1186/s12886-019-1211-1

**Published:** 2019-10-07

**Authors:** Xin Rong, Yu Cai, Mei Li, Yuan Fang, Tian Tian, Yingzi Pan

**Affiliations:** Department of Ophthalmology, Peking University First Hospital, 8 Xi Shi Ku Street, Xi Cheng District, Beijing, 100034 People’s Republic of China

**Keywords:** Peripapillary retinal artery, Normal tension glaucoma, Discrimination accuracy

## Abstract

**Background:**

Glaucoma, an important cause of visual impairment in many countries, remains a common eye condition due to difficulties in its early diagnosis. We analyzed the characteristics of retinal arteries to add a valuable technology for helping the normal tension glaucoma (NTG) diagnosis.

**Methods:**

This study included 51 patients with newly diagnosed NTG with hemifield defects and 60 age-matched controls. Peripapillary retinal arteriolar calibers (PRACs) photoed by non-mydriatic retinal camera were measured using ImageJ by two masked readers. We also performed spectral-domain optical coherence tomography to evaluate retinal nerve fiber layer thickness (RNFLT) and optic disc parameters. Their relations to retinal arteriolar calibers were investigated by univariate and multivariate linear regression. The area under the receiver operating characteristic curve (AUROC) was used to confirm the powers to detect NTG by PRACs.

**Results:**

PRACs in four quadrants were significantly reduced in individuals with first diagnosed NTG (82 ± 15.1 μm, 80 ± 13.6 μm, 71 ± 11.6 μm, and 64 ± 10.0 μm) compared with those in age-matched controls (101 ± 9.8 μm, 105 ± 8.7 μm, 90 ± 7.5 μm, and 82 ± 9.8 μm). Superotemporal and inferotemporal PRACs in the visual field-affected hemifield were narrower than those in the unaffected hemifield in NTG group (*P* ≤ 0.004). Temporal PRACs in the RNFL unaffected hemifield were significantly narrower than in healthy eyes (*P* < 0.001). Superotemporal PRAC showed a significant correlation with superior RNFLT (β = 0.659, *P* < 0.001), and a similar relationship was found between inferotemporal PRAC and inferior RNFLT (β = 0.227, *P* = 0.015). The diagnostic capability of temporal PRACs was satisfactory (superotemporal PRAC; AUROC 0.983, cut-off value 84.7 μm, inferotemporal PRAC; AUROC 0.946, cut-off value 94.2 μm).

**Conclusions:**

PRAC and inferotemporal PRAC are valid parameters for discriminating patients with NTG.

## Background

According to the World Health Organization, as an eye disease known for centuries, glaucoma remains on the public health agenda due to difficulties in its early diagnosis [1]. Objective evidence of early glaucoma lies in retinal ganglion cells loss and optic nerve head damage associated with visual field defects. Thus, measuring the retinal nerve fiber layer and visual field examination are the most commonly used methods at present. The evaluation of retinal nerve fiber layer (RNFL) defects requires sufficient practical experience. The visual field, a subjective test, appears abnormal after a significant number of ganglion cell axons (20–40%) are lost [2]. Hence, there is a need for an objective, simple, noninvasive, highly sensitive method to increase the glaucoma detection rate. The vascular theory of glaucoma considers the progressive loss of RGCs is a consequence of insufficient blood supply, and approximately two-thirds of patients with glaucoma have normal intraocular pressure (IOP) at the initial stages [3]. The retinal vessels, which supply the nerve fiber layers, can easily be inspected on fundus photos; therefore, we assessed retinal vessel caliber features and evaluated its detection accuracy for patients with first diagnosed normal tension glaucoma (NTG).

## Methods

### Study design and population

This hospital-based, cross-sectional case control study was conducted from January 2016 to June 2017. This research adhered to the Declaration of Helsinki and was approved by the local ethics committee of the Institutional Review Board of Peking University First Hospital. We obtained informed consent for participation from all subjects. Newly confirmed NTG cases and healthy age-matched controls were recruited during the same period from Peking University First Hospital in Beijing, China, and all diagnoses were made by glaucoma experts.

We extracted basic demographic features (age, sex, race, and preexisting medical conditions); performed complete routine ophthalmic examination; measured central corneal thickness (CCT) by A-scan ultrasound pachymetry (SW-2100, Sowei, Tianjin); and obtained stereoscopic optic disc photographs centered on the disc with a digital color retinography (EOS 50D CMOS, Canon CR-2, Tokyo, Japan) and a 3.5-mm-diameter circle centered on the optic disc with spectral-domain optical coherence tomography (Optovue, Fremont, CA, USA); and 24–2 Humphrey Swedish Interactive Threshold Algorithm (SITA) standard VF testing (Humphrey Field Analyzer, Carl Zeiss Meditec, Dublin, CA, USA) for all participants.

### Eligibility criteria

The patients with NTG demonstrated reproducible visual field loss consistent with neuroretinal rim narrowing or excavation and nerve fiber layer defects. The diurnal IOP curve showed the highest intraocular pressure ≤ 21 mmHg. No subjects had abnormal anterior chamber angles on gonioscopy or secondary causes of optic nerve damage on posterior segment examination.

Controls had IOP ≤ 21 mmHg, cup disc ratio ≤ 0.6, and cup disc ratio asymmetry ≤0.2. Normal slit lamp examinations, normal optic nerves with no rim or RNFL changes and normal visual field were also confirmed in controls. Only age-matched control subjects without a family history of glaucoma were included.

Reliable perimetry was defined as a fixation loss rate ≤ 15% and false positive and negative rates ≤15%. A signal strength index > 50 was used to define good quality OCT images. An important inclusion criterion for cases and controls was subjects with refractive error between − 6 D and + 3 D and a cylinder correction within ±3 D, and axial length < 26 mm, because over refraction cause discrepancies in photographic magnification and affect the accuracy of retinal vessel measurement [4, 5]. We excluded subjects with diabetes, hypertension, hyperlipemia, cardiac-cerebral abnormalities, migraine, any autoimmune connective tissue disease, history of anti-glaucoma medications, history of intraocular or laser surgery, eye trauma, and older than 70 years because these events may contribute to vascular structural changes [6]. This research only included mild and moderate (visual field MD ≥ − 12 dB) NTG eyes with single-hemifield involvement, which means superior or inferior visual field loss consistent with nerve fiber layer defects on fundus photography.

### Retinal arteriole caliber measurements

Professional technicians used a digital non-mydriatic retinal camera to acquire images centered on the optic disc and covering the optic cup per patient and stored them at 2592*1728 pixels in size (the actual size of the image is 22.3*14.9 mm) on a computer. Then, these images were forwarded to a drive where they were analyzed by 2 glaucoma-trained masked ophthalmologists.

**Fig. 1:**
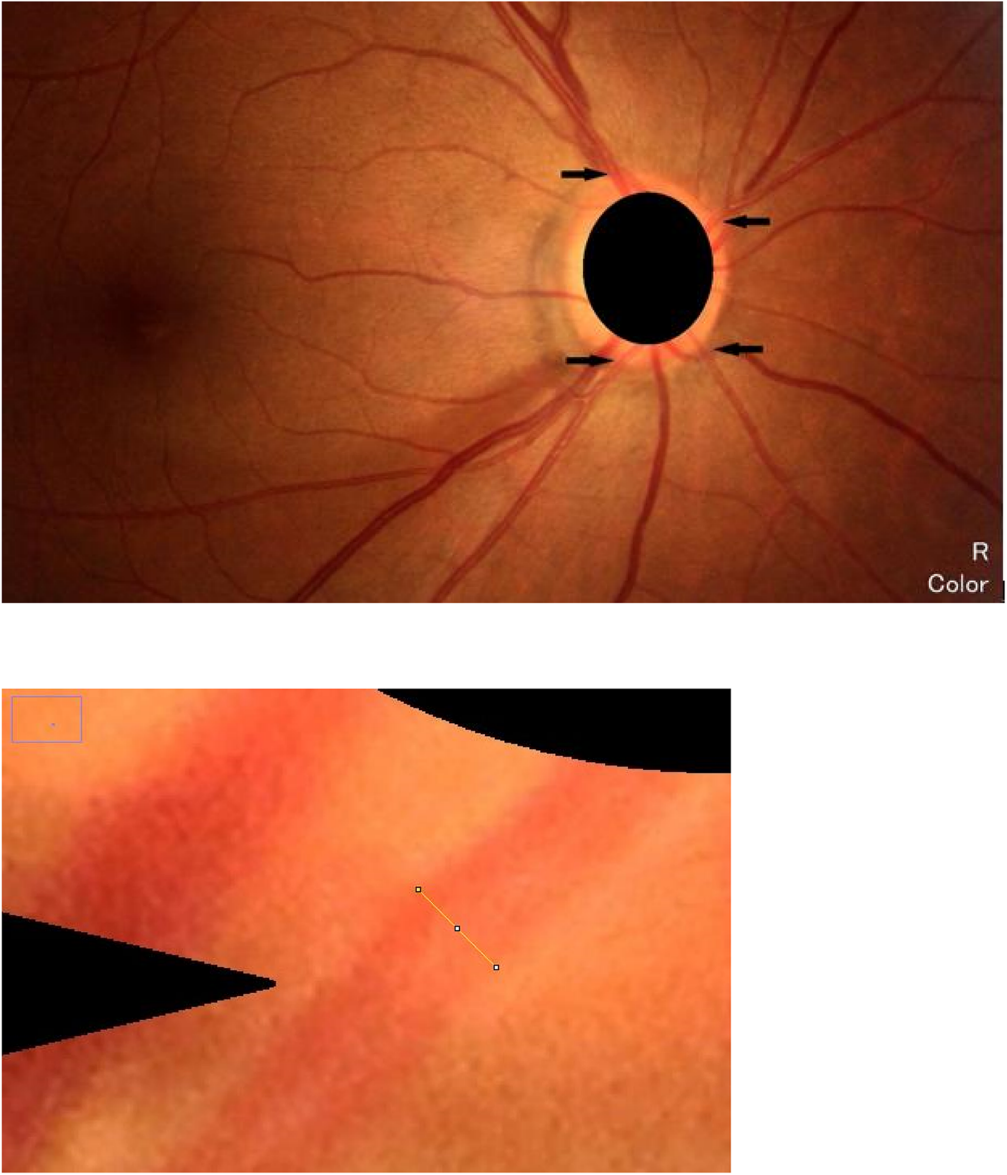
Fundus photograph showing method of measurement of peripapillary retinal arteriolar calibers (PRACs). We plotted superior temporal, inferior temporal, superior nasal, and inferior nasal retinal arteries (black arrows), and then measured their calibers at the optic disc border (yellow line) using Image J software

Photographs with poor quality or any one of four main retinal arteriole branches within the disc were excluded. The retinal arteries are bright red, with obvious reflection, and the branches are inclined at an acute angle. While, retinal veins are dark red, the reflection is not obvious, and the branches are obtuse. Normal arteriovenous ratio is 2:3 [7]. We plotted superior temporal, inferior temporal, superior nasal, and inferior nasal retinal arteries and then measured their calibers at the optic disc border using ImageJ (National Institutes of Health, Bethesda, MD), as shown in Fig.1.

The measurement protocol was as follows:

1. The original full size version of a received image was opened using ImageJ.
2. A scale bar was set for this image. Since the amplification of a Canon CX-1 fundus camera is 1.1, 2592 pixels equals 20,273 μm. We entered 2592 for the distance in pixels, 20,273 for the known distance, and micron for the unit of length.
3. The image was magnified 200 times. Two ophthalmologists masked to diagnosis evaluated four parapapillary retinal arteriole calibers (PRACs) per eye, which were measured five times on each artery with the average recorded. Both observers measured all images two times to assess interrater reliability.

### Optical coherence tomography measurements

RTVue-OCT was used according to the standard glaucoma protocol, and each eye underwent a three-dimensional optic disc scan. The main parameters of the ONH scan used in this research were the average RNFL thickness (RNFLT), superior RNFL thickness, inferior RNFL thickness, cup area, rim area, and cup/disc area ratio.

### Statistical methods

All statistical analyses were performed in SPSS version 20.0. Differences in NTG vs controls and cases with superior RNFL defects vs inferior RNFL defects were tested by using independent two-tailed Student’s t-tests for continuous variables and χ^2^ tests for categorical variables. We used repetitive measurement and analysis of variance to compare four PRACs for each group.

In case-only analyses, univariate and multiple linear regression analyses were used to assess associated factors for PRACs. Pearson’s correlation test was performed using factors of age, sex, CCT, cup area, rim area, cup/disc area ratio, RNFL thickness and visual field global indices for four PRACs. Multivariate analyses were executed in an enter approach, and standardized beta coefficient (β) and *P* values are reported. The area under the receiver operating characteristic curve (AUROC) was used to compare the powers to detect NTG in the parameters of the PRAC, RNFL thickness and mean deviation (MD) read in Humphrey perimetry.

Interclass correlation coefficients obtained by the two-way mixed-effect model showed good interrater and intrarater reliability (intrarater ICC ≥ 0.993 and interrater ICC ≥ 0.973; Table 1). All statistical tests were two sided, and a *P* value < 0.05 was considered statistically significant.

**Table 1:** Internal consistency and test-retest reliability of PRAC

	Internal correlation coefficient (95% CI)		Intraclass correlation coefficient (95% CI)	
	NTG group	Normal group	NTG group	Normal group
	(*n* = 102)	(*n* = 120)	(*n* = 102)	(*n* = 120)
Superotemporal PRAC	0.998 (0.994–0.999)	0.995 (0.981–0.999)	0.978 (0.952–0.990)	0.983 (0.940–0.995)
Inferotemporal PRAC	0.995 (0.989–0.998)	0.995 (0.928–0.999)	0.975 (0.946–0.989)	0.976 (0.915–0.993)
Superonasal PRAC	0.996 (0.992–0.998)	0.996 (0.984–0.999)	0.982 (0.961–0.992)	0.973 (0.906–0.992)
Inferonasal PRAC	0.996 (0.991–0.998)	0.993 (0.975–0.998)	0.980 (0.956–0.991)	0.975 (0.912–0.993)

Two-way mixed-effect model

## Results

**Fig. 2:**
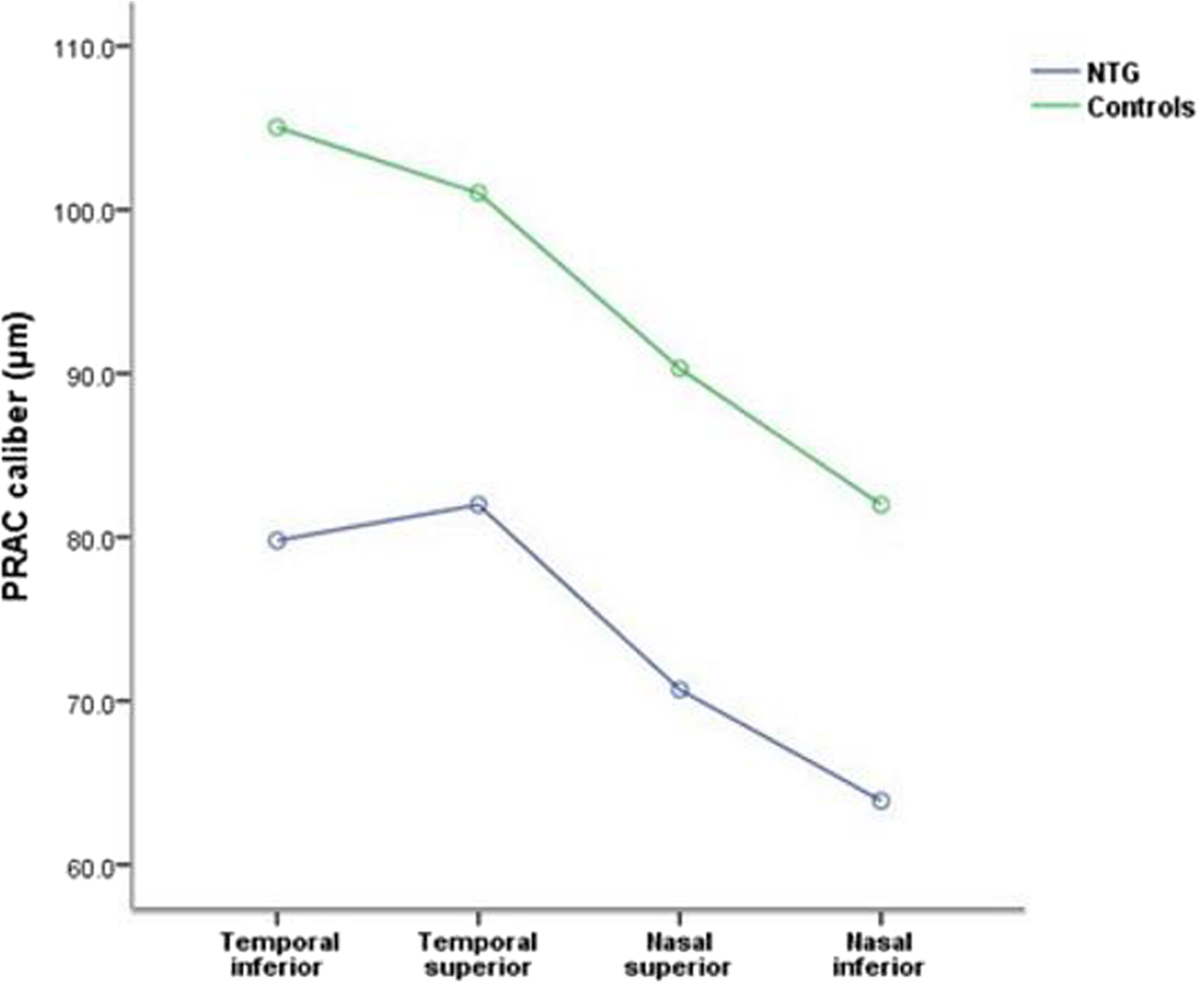
The sort order of mean PRAC caliber in four quadrants in NTG patients and controls

Our study ultimately included 51 eyes (30 male and 21 female) in the NTG group and 60 eyes (30 male and 30 female) in the control group. Randomly selected data from one eye per individual, IOP at the date of retinal photography were used for analysis. No age, gender (χ2 = 0.864, *P* = 0.353),IOP or CCT differed between the two groups (Table 2). PRACs in four quadrants, RNFLT, cup area, rim area, cup/disc area ratio, and MD in patients with NTG were significantly different from those in controls (*P* < 0.0001; Table 2). Of these measurements, PRAC was more narrow in all quadrants in cases than in controls (18.6% reduced for superior temporal, 23.8% reduced for inferior temporal, 21.1% reduced for superior nasal, 14.6% reduced for inferior nasal, respectively). In addition, PRAC in normal eyes followed temporal inferior > temporal superior > nasal superior > nasal inferior, while the rules were not followed by NTG eyes (P < 0.0001; Fig.2). Compared with controls, subjects with NTG had thinner RNFL in both superior and inferior hemifields (both *P* < 0.001; Table 2).

**Table 2:** Baseline characteristics of the study subjects

Variable	Controls (*n* = 60)	NTG (*n* = 51)	t values	*P* values
Age, mean (SD), y	59 (10.5)	54 (12.1)	−1.703	0.093*
Sex, n (%)				0.353**
Female	30 (50%)	21 (41%)	/	/
male	30 (50%)	30 (59%)	/	/
IOP, mean (SD), mm Hg	14 (2.0)	15 (1.6)	1.110	0.271*
CCT, mean (SD), μm	540.6 (17.8)	532.7 (24.4)	−1.398	0.169*
MD, mean (SD),dB	−2 (1.0)	−6 (4.6)	−5.992	< 0.001*
Cup area, mean (SD),mm^2^	1.01 (0.53)	1.53 (0.58)	3.653	< 0.001*
Rim area, mean (SD),mm^2^	1.56 (0.34)	0.75 (0.34)	−9.411	< 0.001*
Cup/disc area ratio,mean (SD)	0.34 (0.18)	0.65 (0.18)	6.107	< 0.001*
Avg.RNFL, mean (SD), μm	116 (8.9)	85 (11.1)	−11.642	< 0.001*
Sup.RNFL, mean (SD), μm	117 (8.6)	88 (14.7)	−8.847	< 0.001*
Inf.RNFL, mean (SD), μm	115 (10.7)	82 (12.0)	−11.103	< 0.001*
Superotemporal PRAC, mean (SD), μm	101 (9.8)	82 (15.1)	−5.538	< 0.001*
Inferotemporal PRAC, mean (SD), μm	105 (8.7)	80 (13.6)	−8.156	< 0.001*
Superonasal PRAC, mean (SD), μm	90 (7.5)	71 (11.6)	−7.417	< 0.001*
Inferonasal PRAC, mean (SD), μm	82 (9.8)	64 (10.0)	−7.252	< 0.001*

*Avg.RNFL* average RNFL thickness, *Sup.RNFL* superior RNFL thickness;Inf.RNFL, inferior RNFL thickness

*Independent t-test. ** χ^2^ test

NTG cases were stratified by the location of RNFL defects. Table 3 shows the characteristics of NTG patients with superior or inferior RNFL loss. All four PRACs in these two NTG subgroups were narrower than those in the healthy eye group; these changes were not limited to the affected hemifield and appeared in another hemifield (*P* < 0.001 for all comparisons; Table 3). The two glaucoma subtypes did not vary significantly in age, IOP, optic disc clinical features, and MD (*P* ≥ 0.195; Table 3). Moreover, temporal PRACs in the RNFL unaffected hemifield were wider than in the affected hemifield (*P* ≤ 0.004; Table 3), While narrower than normal eyes (*P* < 0.001; Table 3). Nasal PRACs showed no significant differences (*P* ≥ 0.257; Table 3).

**Table 3:** Descriptive analysis of clinical characteristics by the location of RNFL defects on fundus

	Superior RNFLD (*n* = 17)	Inferior RNFLD (*n* = 34)	*P1*	*P*2	*P3*
Age, mean (SD), y	55 (11.6)	53 (12.5)	0.601	0.297	0.082
IOP, mean (SD), mm Hg	15 (1.3)	15 (1.8)	0.545	0.230	0.445
CCT, mean (SD), μm	530.6 (16.4)	533.7 (27.7)	0.669	0.078	0.301
MD, mean (SD),dB	− 5 (4.2)	−7 (4.8)	0.195	< 0.001	< 0.001
Cup area, mean (SD),mm^2^	1.57 (0.69)	1.51 (0.53)	0.740	< 0.001	< 0.001
Rim area, mean (SD),mm^2^	0.69 (0.30)	0.78 (0.35)	0.326	0.006	< 0.001
Cup/disc area ratio, mean (SD)	0.67 (0.18)	0.65 (0.18)	0.684	< 0.001	< 0.001
Sup.RNFL, mean (SD), μm	76 (8.4)	94 (13.4)	< 0.001	< 0.001	< 0.001
Inf.RNFL, mean (SD), μm	89 (11.7)	78 (10.8)	0.003	< 0.001	< 0.001
Superotemporal PRAC, mean (SD), μm	72 (11.7)	87 (14.2)	< 0.001	< 0.001	< 0.001
Inferotemporal PRAC, mean (SD), μm	87 (10.7)	76 (13.5)	0.004	< 0.001	< 0.001
Superonasal PRAC, mean (SD), μm	69 (7.4)	71 (13.3)	0.618	< 0.001	< 0.001
Inferonasal PRAC, mean (SD), μm	62 (8.6)	65 (10.5)	0.257	< 0.001	< 0.001

Independent t-test. *P1*,*P*-value for Superior RNFLD vs Inferior RNFLD; *P2*,*P*-value for Superior RNFLD vs Controls; *P3,P*-value for Inferior RNFLD vs Controls

Correlation analysis was performed to examine the relationship of PRAC with age, gender, CCT, cup area, rim area, cup/disc area ratio, RNFLT, and visual field global indices in NTG eyes. Using the Pearson correlation coefficient, superonasal and inferonasal PRACs showed a narrowed trend with increasing age(*r* = − 0.359, *P* = 0.010; *r* = − 0.283, *P* = 0.044), whereas temporal PRACs and age were not related. PRACs in all quadrants were not correlated with IOP, CCT or optic disc parameters. As superotemporal PRAC reduced, superior RNFLT exhibited a decreasing trend (*r* = 0.449, *P* = 0.001). Additionally, inferotemporal PRAC and inferior RNFLT had a positive correlation (*r* = 0.369, *P* = 0.008).

We used superotemporal, inferotemporal, superonasal, and inferonasal PRACs as dependent variables. The independent variables included age, gender, CCT, cup/disc area ratio, superior RNFLT, inferior RNFLT and MD. All variables were loaded by the block and enter method. The reductions in superotemporal PRAC and inferotemporal PRAC were significantly associated with decreases in superior RNFLT and inferior RNFLT, respectively (β = 0.659, *P* < 0.001 and β = 0.227, *P* = 0.015). No relation of nasal PRACs with RNFLT was shown in the multivariate analyses (Table 4).

**Table 4:** Multivariable linear regression of RNFL thickness, Cup/disc area ratio and MD value in relation to PRAC in glaucoma case

Variables	Superotemporal PRAC	Inferotemporal PRAC	Superonasal PRAC	Inferonasal PRAC
	*β*(*P-value*)	*β*(*P-value*)	*β*(*P-value*)	*β*(*P-value*)
Age,y	- 0.108 (0.321)	- 0.119 (0.355)	- 0.320 (< 0.001)	- 0.223 (0.013)
sex	0.093 (0.358)	- 0.230 (0.819)	0.064 (0.500)	0.066 (0.492)
CCT, μm	0.085 (0.360)	0.021 (0.824)	0.059 (0.505)	0.059 (0.509)
MD, dB	- 0.004 (0.970)	- 0.031 (0.774)	- 0.113 (0.278)	- 0.079 (0.452)
Cup/disc area ratio	- 0.184 (0.109)	- 0.191 (0.096)	- 0.175 (0.110)	- 0.194 (0.074)
Superior RNFLT	0.659 (< 0.001)	0.158 (0.246)	0.149 (0.142)	0.062 (0.499)
Inferior RNFLT	0.156 (0.323)	0.227 (0.015)	0.131 (0.384)	0.053 (0.727)

*β*, standardized regression coefficient, *RNFLT* retinal nerve fibre layer thickness

**Fig. 3:**
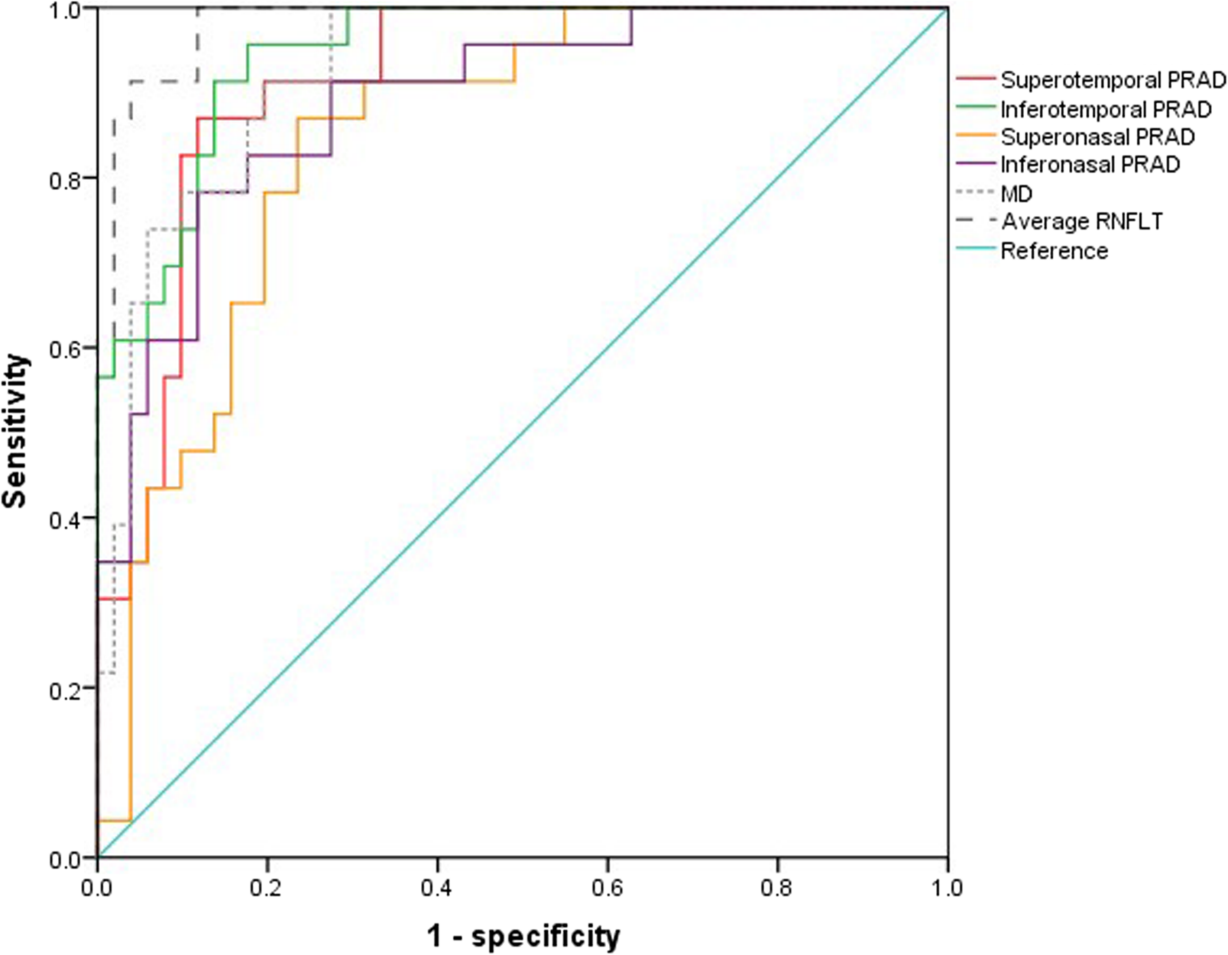
The discriminatory ability of PRAC between NTG patients and healthy controls was analyzed using ROC curves

As shown in Fig. 3, the AUROC of average RNFL thickness, inferotemporal PRAC, MD, superotemporal PRAC, inferonasal PRAC and superonasal PRAC was 0.983, 0.946, 0.929, 0.915, 0.890, and 0.847, respectively. The cut-off value of inferotemporal PRAC was 94.2 μm with a sensitivity of 95.7% and specificity of 84.2%, and the cut-off value of superotemporal PRAC and the corresponding sensitivity and specificity were 84.7 μm, 82.6 and 90.2%, respectively.

## Discussion

This study demonstrates that PRACs in four quadrants were markedly reduced in patients with first diagnosed NTG compared with those in age-matched controls. Additionally, the temporal PRAC in the visual field-affected hemifield was narrower than that in the unaffected hemifield in age-matched and severity-matched NTG. However, this difference was not present in nasal PRAC. There was a strong negative association between temporal PRAC and RNFLT in the corresponding hemifield in eyes with NTG. This correlation remained true after adjusting for age, gender, cup/disc area ratio, and MD values. Furthermore, the diagnostic ability of superotemporal and inferotemporal PRACs were comparable to that of RNFL thickness or MD values in detecting early-stage NTG.

Similar to our results, most previous reports indicated that retinal vessel diameters in NTG, particularly retinal arteriolar diameters, were significantly smaller than those in controls [8–11]. Although few researchers have found no differences, the following points may help explain the discrepancies among these findings. The first point is different types of glaucoma and various stages of patients. Some studies compared all enrolled glaucomatous eyes with nonglaucomatous eyes [9, 11–13], and other researchers did not distinguish patients with NTG from those with POAG [14]. Different types of glaucoma have unique glaucoma-related changes in their mechanical properties. As a subtype of POAG, in NTG, vascular factors may play a more significant role than those in glaucoma with high IOP [15]. The second factor is patients’ systemic condition. Large numbers of studies did not consider connective tissue and vascular diseases, such as diabetes, hypertension, hyperlipemia, cardiac-cerebral abnormalities, migraine and autoimmune connective tissue diseases. All of these diseases have been linked to retinal vascular diameters [16–20]. Moreover, ocular hypotensive drugs such as beta-blocker medications have vasodilating effects, leading to underestimation of the association between glaucoma and retinal arteriolar narrowing [21]. Consequently, our study focused on patients with first diagnosed and untreated NTG. We also strictly excluded patients with the vascular or connective tissue diseases mentioned above.

The third factor is the different locations and technologies used for retinal arteriolar measurements. There were different places used in former articles to measure the vascular diameters. Retinal arterioles usually have branches after go outside the optic disc. In order to reduce heterogeneity, we measured the PRAC at the optic disc border and any subjects with retinal arteriole branching within the disc were excluded. In recent years, semiautomated computer-assisted programs, such as Optimate (Department of Ophthalmology and Visual Science, University of Wisconsin—Madison) and IVAN (Department of Ophthalmology and Visual Science, University of Wisconsin—Madison) software, have been developed to measure average retinal vessel widths in terms of central retinal vessel equivalents. The rationale behind our method was that automatic systems for the assessment of retinal vessels were unavailable for examiners while performing retinography. Our method using ImageJ showed good to excellent interrater and intrarater reliability. Moreover, the nasal retinal vessel may show little or no change in the early stage of glaucoma; thus, the average caliber of retinal vessels may hide some information. In recent years, optical coherence tomography angiography has become a modern instrument for glaucoma patients, mainly because radial peripapillary capillaries (RPCs) have been recognized as a specialized vasculature that supplies the ganglion cell axons [22]. RPC is located in the most superficial retinal, and this layer is perfused by the central retinal arteries. Therefore, our group speculated NTG eyes may have retinal artery abnormalities. If the abnormalities exist in early-stage glaucoma eyes, PRAC opens the possibility of low-cost analysis for glaucoma screening, particularly within epidemiologic studies and primary hospitals.

The chicken–egg correlation between narrowed vessel calibers and glaucomatous optic nerve head damage remains uncertain. RNFL thickness is a marker of optic neuropathy, and some findings indicate that the thinned RNFL lowers the metabolic demands of the retinal tissue, leading to arterial caliber decrease. Conversely, the reverse hypothesis is that RGCs loss is a consequence of insufficient blood supply [17, 23, 24]. Therefore, our study specifically included NTG eyes with hemifield involvement in the hope that this study will aid in understanding the vascular theory of glaucoma pathogenesis. In the present study, NTG eyes had significantly smaller retinal arterioles in the corresponding RNFL defect hemifield than in the RNFL unaffected hemifield, and the latter was narrower than that in healthy eyes. Additionally, the results demonstrated a strong relationship between temporal PRACS and the RNFL thickness of the corresponding hemifield. This is in line with the close association of vascular autoregulatory dysfunction in the retina with glaucoma [24, 25]. Therefore, it is reasonable to propose that narrowing temporal PRAC may be regarded as one of the early risk markers for NTG patients. Our ROC analysis also supported this speculation, that is, both superotemporal and inferotemporal PRACs had a strong ability to distinguish between NTG and normal eyes and were at least similar to RNFL thickness and MD values. Thus, the estimation method we used to analyze retinal arteries may be used clinically to help predict NTG in the early stage.

Several limitations of our study merit discussion. First, this is a cross-sectional study. We found that superotemporal PRAC and inferotemporal PRAC are valid parameters for discriminating patients with NTG. The data can only be used to determine relationships between the features of PRAC and RNFLT. The further perspective study is needed before we can judge the causality of these factors. Second, we did not perform a complete physical examination for every enrolled subject. Instead, medical histories were collected, and base on these we excluded subjects with vascular diseases. Additionally, The PRAD measurements were not determined by Littmann’s formula. This is the widely method used to correct the magnification of a camera, but the telecentric camera applied for this formula is different from the camera used in this study [4, 5]. In order to reduce the error that may be caused by diopter, we excluded subjects with high myopia, high hypermetropia or axial length ≥ 26 mm. Another limitation lies in brachial artery blood pressure, which was not measured while fundus photographs were performed. Hao et al. considered vessel caliber to have no significant changes in the pulse cycle, suggesting that this issue might not influence the results [26]. Third, we employed subjective methods for the measurement of retinal vessel calibers, but good interrater and intrarater reliability confirmed its accuracy. Our result of narrowed retinal arteries in glaucoma was consistent with recent findings using computer-assisted programs.

This research also has several notable strengths. To the best of our knowledge, this study is the first performed in patients with first diagnosed NTG. Graders masked to glaucoma case status measured PRACs, reducing the chance of reader bias. We excluded subjects with vascular diseases or autoimmune connective tissue disease and those with any history of glaucoma therapy, minimizing the chance that the association between PRAC and NTG was the result of uncontrolled confounders. Moreover, these results imply that PRAC is a convenient, inexpensive and noninvasive technique for detecting NTG.

## Conclusions

We reported a promising method that has the potential for routine use in screening patients with NTG. Superotemporal and inferotemporal PRACs are likely to narrowed early than RNFL damage detected by fundus photos. When superotemporal PRAC narrowed by more than 84.7 μm or inferotemporal PRAC narrowed by more than 94.2 μm in one patient, this change deserves our attention and even activates a glaucomatous alarm. The present study also adds to the growing evidence that NTG pathogenesis has an avascular component and may provide insights into why IOP lowering treatment slows but does not halt disease progression in many cases.

This was a pilot study, and future directions include a larger sample size with different glaucoma subtypes in different disease stages, with the aim of determining whether this vascular measurement procedure can also be applied to these subtypes. Longitudinal studies are also needed to investigate whether changes in PRAC are involved in or related to progression of the disease.

## Data Availability

The data used to support the findings of this study are available from the corresponding author upon request.

## Abbreviations

AUROC: Operating characteristic curve
IOP: Intraocular pressure
MD: Mean deviation
PRAC: Parapapillary retinal arteriole caliber
RNFL: Retinal nerve fiber layer
RNFLT: Retinal nerve fiber layer thickness
β: Beta coefficient
